# Relationship between the Rumen Microbiome and Residual Feed Intake-Efficiency of Brahman Bulls Stocked on Bermudagrass Pastures

**DOI:** 10.1371/journal.pone.0091864

**Published:** 2014-03-18

**Authors:** Joshua C. McCann, Leanne M. Wiley, T. David Forbes, Francis M. Rouquette, Luis O. Tedeschi

**Affiliations:** 1 Department of Animal Science, Texas A&M University, College Station, Texas, United States of America; 2 Texas A&M Agrilife Research, Uvalde, Texas, United States of America; 3 Texas A&M Agrilife Research, Overton, Texas, United States of America; Technion-Israel Institute of Technology Haifa, Israel

## Abstract

Residual feed intake (**RFI**) testing has increased selection pressure on biological efficiency in cattle. The objective of this study was to assess the association of the rumen microbiome in inefficient, positive RFI (**p-RFI**) and efficient, negative RFI (**n-RFI**) Brahman bulls grazing ‘Coastal’ bermudagrass [*Cynodondactylon* (L.) Pers.]under two levels of forage allowance (high and low stocking intensity). Sixteen Brahman bulls were previously fed in confinement for 70 d to determine the RFI phenotype. Bulls were then allotted 60 d stocking on bermudagrass pastures to estimate RFI using the n-alkane technique. At the conclusion of the grazing period, rumen liquid samples were collected from each bull by stomach tube to evaluate the rumen microbiome. Extraction of DNA, amplification of the V4-V6 region of the 16S rRNA gene, and 454 pyrosequencing were performed on each sample. After denoising the sequences, chimera checking, and quality trimming, 4,573 ± 1,287 sequences were generated per sample. Sequences were then assigned taxonomy from the Greengenes database using the RDP classifier. Overall, 67.5 and 22.9% of sequences were classified as *Bacteroidetes* and *Firmicutes*, respectively. Within the phylum *Bacteroidetes*, *Prevotella* was the most predominant genus and was observed in greater relative abundance in p-RFI bulls compared with n-RFI bulls (*P* = 0.01). In contrast, an unidentified *Bacteroidales* family was greater in relative abundance for n-RFI bulls than p-RFI (26.7 vs. 19.1%; *P* = 0.03). *Ruminococcaceae* was the third most abundant family in our samples, but it was not affected by RFI phenotype. No effect of stocking intensity was observed for bacterial taxa, but there was a tendency for alpha diversity and operational taxonomic unit richness to increase with lower stocking intensity. Results suggested the rumen microbiome of p-RFI Brahman bulls has greater levels of *Prevotella*, but the bacterial community composition was unaffected by stocking intensity.

## Introduction

Increasing feed and land costs are the greatest variable costs in beef cattle production [1]. Decreasing cattle maintenance requirements through more efficient feed utilization may significantly influence total costs. Improvements in efficiency independent of phenotypic performance can be described by residual feed intake (**RFI**) [2], [3]. Evaluation of RFI is often measured in confinement (**RFIc**); however, there is limited research related to RFI for grazing cattle (**RFIg**)where it may potentially be more useful to the industry [4]–[6].

The RFI has been correlated to lower heat production and methane emissions, and greater diet digestibility [2]. Animals with a negative RFI (**n-RFI**) have lower DMI than expected and may be classified as efficient; whereas less efficient, positive RFI animals (**p-RFI**) have greater feed intake at the same level of production. Digestibility and fermentation account for 19% of the variation in RFI [2] indicating rumen microbial populations may be linked to observed RFI phenotypes. Differential gradient gel electrophoresis has demonstrated different banding patterns between the microbiome of p-RFI and n-RFI steers [7].

The use of DNA-based, high-throughput sequencing technologies provides a more encompassing view of the rumen microbiome though few efforts have been made to associate the rumen microbiome with RFI phenotypes. Large bacterial community changes in the rumen bacteria were not observed in dairy cows from divergent RFI phenotypes [8]. However, the cows were ranked as heifers for RFI at 8 mo of age and the trial did not occur until 3 years of age. Greater organic matter digestion and ruminal ammonia have been observed for *Bos indicus* cattle compared with *Bos Taurus* [9]. The objective of this study was to evaluate the rumen microbiome of Brahman bulls, selected for divergent RFIc phenotypes, grazing bermudagrass pastures at high or low stocking intensity.

## Materials and Methods

### Experimental design

The experimental protocol was approved by the Institutional Animal Care and Use Committee at Texas A&M University (AUP 2010–053). This study was conducted at the Texas A&MAgriLife Research and Extension Center in Overton, Texas, in the Pineywoods vegetation zone in east Texas (32°16′N 94°59′W, with an average rainfall of 100 cm, and mean temperature of 30.15°C) during the summer of 2010. Purebred Brahman bulls (n = 33) were measured for ADG and DMI in confinement. The bulls were fed a commercially available growing ration(65% TDN, 2.35 Mcal/kg ME, and 11% CP) in confinement using Calan gates at 2.8% BW. This % BW level was assessed to be 98–100% of the daily intake but with limited to no orts. The most efficient (n-RFI; n = 8) and inefficient (p-RFI; n = 8) bulls were separated into four groups with two p-RFI and two n-RFI bulls per group. They were randomly allotted into two replicate Coastal bermudagrass [*Cynodondactylon*(L.) Pers.]pastures(18.9% CP, 68.8% NDF, and 2.1% starch)at high or low stocking intensity (**HSTK** and **LSTK**, respectively) for 60 d. Stocking intensity (**SI**) was quantified and expressed by forage allowance (**FA**). Forage allowance was calculated by dividing total forage DM by the collective total animal BW per unit area of land. A single FA was calculated for each stocking rate per year.This “relationship between animal live weight and forage mass per unit area of the specific unit of land being grazed at any one time” is referred to as grazing pressure [10].The bulls (initial BW for n-RFI = 379±61.6 kg, p-RFIg = 422±18.6 kg) were weighed bi-weekly throughout the 60 d study during which three, 10-d intake measurement periods were conducted using the n-alkane method [11].

### Microbiome Sampling and Processing

At the conclusion of the 60 day grazing period, rumen contents were aspirated with a flexible tube inserted orally into the rumen. The contents of the rumen were thoroughly mixed and 2 samples of 400 ml were collected and frozen at −20°C. The DNA was extracted from rumen content samples using QIAamp stool DNA mini kit (Qiagen, Valencia, CA) as previously described [12]. Amplification of the V4–V6 segment of 16S rRNA gene utilized barcoded primer tags and the universal eubacterial primers 530F (5′-GTGCCAGCMGCNGCGG-3′) and 1100R (5′-GGGTTNCGNTCGTTG-3′) as previously described [13].Pyrosequencing was performed using a Genome Sequencer FLX System (Roche, Branford, CT) with Titanium chemistry at Research and Testing Laboratory (Lubbock, TX).

### Sequence read analysis

Sequences for each sample were processed using the QIIME pipeline v1.5.1 [14].Initially, raw flowgrams for sequences were denoised, demultiplexed, and truncated at 415 bp using AmpliconNoisewith Perseus option for de novo chimera removal [15].Denoising accounted for error associated with pyrosequencing and PCR, while truncation was performed when the average base pair quality score <25. Clustering of operational taxonomic units (**OTUs**) at 97% similarity with UCLUST generated 4,906 OTUs with an average of 998 OTUs per sample [16]. Cluster representative sequences were aligned to the Greengenes database (gg_12_10_otus; secondgenome.com) with PyNAST [17]. The RDP Classifier assigned taxonomic classification using a 0.8 bootstrap value based on near full-length sequences in the Greengenes database [18], [19]. Singleton OTUs were removed to prevent artificial diversity inflation [20]. After sample size standardization to smallest library size (1,800 sequences), OTU richness, alpha, and beta diversity were estimated. Metrics used include: observed OTUs, ACE,and Shannon [21], [22]. Sequences are accessible on the MG-RAST website under accession numbers4549225.3, 4549226.3, 4549227.3, 4549228.3, 4549229.3, 4549230.3, 4549231.3, 4549232.3, 4549233.3, 4549234.3, 4549235.3, 4549236.3, 4549237.3, 4549238.3, 4549239.3, 4549240.3.

### *Statistical analysis*

Relative abundances of bacteria present >1% at phylum, family, or genus taxonomic level were logit transformed [z = log(p/(1-p))] to normally distribute residuals, where *p* represented the relative abundance of a bacterial taxa. Transformed data was analyzed using the GLIMMIX procedure of SAS 9.3 (SAS Inst. Inc., Cary, NC). Terms in the model included stocking intensity, RFI category, and stocking intensity × RFI category, with stocking intensity nested within pasture included as a random effect. Treatment means were calculated using the LSMEANS option and back transformed [p = 10^z^/(1+10^z^)].

## Results

### OTU richness, alpha and beta diversity

After pyrosequencing of 16 samples, 73,178 high-quality reads were used for downstream analysis and clustering of sequences yielded 4,906 OTUs at 97% similarity. Over 99% of OTU sequences aligned to phyla in the Greengenes database, while 72 and 43% of sequences were assigned at the family and genus taxonomic level, respectively.After rarefaction to 1,800 sequences per sample, greater OTUs were observed for bulls grazing LSTK bermudagrass pastures (Table 1; *P* = 0.06). An additional richness metric, ACE, also indicated a tendency for OTU richness to increase (*P* = 0.10) 122% for bulls grazing LSTK pastures compared with HSTK. The tendency to increase in observed OTUs with LSTK suggested the increase in alpha diversity (Shannon index) was due to greater OTU richness. No difference was observed in OTU richness or Shannon index between n-RFI and p-RFI bulls (*P*≥0.14). Comparison of microbiome communities by principal coordinate analysis did not reveal patterns related to RFIc phenotype or stocking intensity (data not shown).

**Table 1:** Effect of RFIc and SI on OTU richness and alpha diversity at 97% similarity after rarefaction to 1,800 sequences per sample.^1^

	n-RFI		p-RFI			*P*-value		
Item	High SI	Low SI	High SI	Low SI	SEM^3^	SI	RFIc	RFIc*SI
Shannon^2^	7.8	8.3	6.5	8.4	0.38	0.09	0.14	0.11
ACE	1480.1	1714.6	1299.9	1654.3	96.2	0.10	0.21	0.52
Observed OTUs	605.2	697.0	512.3	695.8	36.1	0.06	0.22	0.23

1 RFIc  =  residual feed intake in confinement; SI  =  stocking intensity; OTU  =  operational taxonomic unit.

2 Shannon index as calculated by Shannon and Weaver (1949).

3 SEM  =  standard error of the mean.

### Effect on bacterial taxa

**Figure 1:**
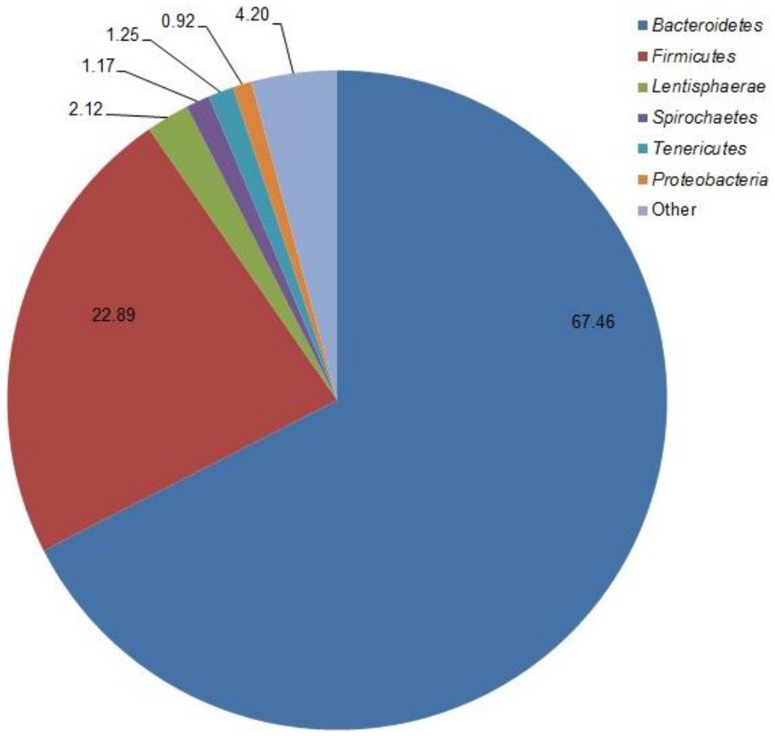
Relative abundance of ruminal bacteria phyla of Brahman bulls at low and high stocking intensities.

Across all samples, *Bacteroidetes* and *Firmicutes* characterized 67.5 and 22.9% of the sequences, respectively (Figure 1). The average *Bacteroidetes*:*Firmicutes* ratio was 2.9∶1. Additional phyla detected including *Lentisphaerae*, *Spirochaetes*, *Tenericutes*, and *Proteobacteria* were less than 5% of sequences in any sample. Phylum *Bacteroidetes* represented the majority of sequences in every treatment ranging from 61.7 (n-RFI, LSTK) to 78.4% (p-RFI, HSTK; Table 2). Phylum *Firmicutes* ranged from 14.6 (p-RFI, HSTK) to 29.7% (n-RFI, LSTK) in relative abundance. There was a tendency for an RFIc×SI interaction for the phylum *Lentisphaerae*(*P* = 0.09). No effects of SI or RFIc were observed at the phylum taxonomic level (*P*≥0.1).

**Table 2:** Effect of RFIc and SI on relative abundance of bacteria phyla.^1^

	n-RFI		p-RFI		*P*-value		
Phyla	High SI	Low SI	High SI	Low SI	SI	RFIc	RFIc*SI
*Bacteroidetes*	66.9	61.7	78.4	62.8	0.35	0.20	0.27
*Firmicutes*	21.3	29.7	14.6	26.0	0.28	0.27	0.62
*Lentisphaerae*	2.8	1.2	2.0	2.4	0.41	0.54	0.09
*Spirochaetes*	1.3	0.9	1.1	1.4	0.91	0.59	0.30
*Tenericutes*	1.2	1.4	0.9	1.5	0.31	0.63	0.42
*Proteobacteria*	1.1	0.9	0.8	0.9	0.89	0.51	0.63

1 RFIc  =  residual feed intake in confinement; SI  =  stocking intensity; relative abundance  =  percent of total bacteria sequences; phyla listed were detected at greater than 1% relative abundance averaged across all samples.

*Prevotellaceae* was the most prevalent identified family observed in all treatments representing more than 19% of all sequences. Greater relative abundance of *Prevotellaceae* was observed in p-RFI bulls (Table 3; *P* = 0.01): a 169% increase compared with n-RFI bulls (33.2 vs. 19.6%). However, n-RFI bulls had an unidentified *Bacteroidales* family (1) in the greatest relative abundance overall and was 140% greater than p-RFI bulls (26.7 vs. 19.1%; *P* = 0.03). Family *Ruminococcaceae* was the third most prevalent family except for the p-RFI bulls on HSTK pastures. *Ruminococcaceae* relative abundance was not affected by SI or RFIc phenotype. Families BS11, *Lachnospiraceae*, *Bacteroidaceae*, *Spirochaetaceae*, as well as four unidentified families were not affected by SI or RFIc phenotype (*P*≥0.20). An RFIc×SI interaction was observed for families *Paraprevotellaceae* (*P* = 0.04) and *Victivillaceae* (*P* = 0.09); both families were observed in greater relative abundance in the HSTK treatment for the n-RFI phenotype bulls and the LSTK treatment for p-RFI bulls. At the genus taxonomic level, all sequences within family *Prevotellaceae*classified as *Prevotella* and were consistent with previously discussed effects (data not shown).

**Table 3:** Effect of RFIc and SI on relative abundance of bacteria families.^1^

	n-RFI		p-RFI		*P*-value		
Families	High SI	Low SI	High SI	Low SI	SI	RFIc	RFIc*SI
*Prevotellaceae*	19.5	19.8	40.5	25.9	0.54	0.01	0.15
Unidentified family 1^2^	25.4	28.0	14.5	23.8	0.17	0.03	0.20
*Ruminococcaceae*	11.7	13.7	8.5	14.1	0.35	0.54	0.46
BS11	3.2	3.9	9.3	3.9	0.57	0.32	0.31
*Paraprevotellaceae*	5.9	2.6	3.2	4.1	0.40	0.74	0.04
*Lachnospiraceae*	2.9	3.9	1.8	4.2	0.20	0.44	0.28
*Victivillaceae*	2.8	1.2	2.0	2.4	0.41	0.54	0.09
*Bacteroidaceae*	1.9	0.9	1.0	0.9	0.54	0.55	0.49
Unidentified family 2^2^	1.6	2.1	1.4	1.6	0.66	0.27	0.77
Unidentified family 3^3^	0.8	1.6	0.5	0.8	0.44	0.33	0.82
Unidentified family 4^4^	1.2	1.1	0.9	1.6	0.61	0.98	0.20
Unidentified family 5^5^	0.9	1.4	0.6	1.1	0.38	0.33	0.77
*Spirochaetaceae*	0.9	0.7	0.8	1.0	0.95	0.64	0.38

1 RFIc  =  residual feed intake in confinement; SI  =  stocking intensity; relative abundance  =  percent of total bacteria sequences; families listed were detected at greater than 1% relative abundance averaged across all samples.

2 Order *Bacteroidales*.

3 Order *Coriobacteriales*.

4 Order *Clostridiales*.

5 Class *Clostridia*.

### Core microbiome

Bacterial taxa present in all samples were considered members of the core microbiome for Brahman bulls grazing bermudagrass pastures. Of the 22 core OTUs, 15 were classified within the phylum *Bacteroidetes* (Table 4). Moreover, 40% of *Bacteroidetes* core OTUs were represented by the *Prevotella* genera. Additional core OTUs included genera *Ruminococcus*, *Succiniclasticum*, and two OTUs from the family *Victivallaceae*. The core microbiome was also determined for n-RFI and p-RFI bulls. Core OTUs observed only in n-RFI bulls included four *Ruminococcaceae* OTUs, five *Prevotella* OTUs, and a single *Paludibacter*OTU (Table S1). We observed ten *Prevotella*, three *Lachnospiraceae*, two *Oscillospira*, and a single *Treponema* OTU in the rumen microbiome of all p-RFI bulls (Table S2).

**Table 4:** Core microbiome OTUs detected in all samples.^1^

Phylum	Class	Order	Family	Genus	Species
*Bacteroidetes*	*Bacteroidia*	*Bacteroidales*	*Paraprevotellaceae*	CF231	
*Bacteroidetes*	*Bacteroidia*	*Bacteroidales*	*Prevotellaceae*	*Prevotella*	*ruminicola*
*Bacteroidetes*	*Bacteroidia*	*Bacteroidales*	*Prevotellaceae*	*Prevotella*	
*Bacteroidetes*	*Bacteroidia*	*Bacteroidales*	*Prevotellaceae*	*Prevotella*	
*Bacteroidetes*	*Bacteroidia*	*Bacteroidales*	*Prevotellaceae*	*Prevotella*	
*Bacteroidetes*	*Bacteroidia*	*Bacteroidales*	*Prevotellaceae*	*Prevotella*	
*Bacteroidetes*	*Bacteroidia*	*Bacteroidales*	*Prevotellaceae*	*Prevotella*	
*Bacteroidetes*	*Bacteroidia*	*Bacteroidales*			
*Bacteroidetes*	*Bacteroidia*	*Bacteroidales*			
*Bacteroidetes*	*Bacteroidia*	*Bacteroidales*			
*Bacteroidetes*	*Bacteroidia*	*Bacteroidales*			
*Bacteroidetes*	*Bacteroidia*	*Bacteroidales*			
*Bacteroidetes*	*Bacteroidia*	*Bacteroidales*			
*Bacteroidetes*	*Bacteroidia*	*Bacteroidales*			
*Bacteroidetes*	*Bacteroidia*	*Bacteroidales*			
*Firmicutes*	*Clostridia*	*Clostridiales*	*Ruminococcaceae*	*Ruminococcus*	
*Firmicutes*	*Clostridia*	*Clostridiales*	*Veillonellaceae*	*Succiniclasticum*	
*Firmicutes*	*Clostridia*	*Clostridiales*			
*Lentisphaerae*	*Lentisphaeria*	*Victivallales*	*Victivallaceae*		
*Lentisphaerae*	*Lentisphaeria*	*Victivallales*	*Victivallaceae*		
*Proteobacteria*	*Alphaproteobacteria*				

1 OTUs  =  operational taxonomic units; taxonomic classification is given at the greatest taxonomic resolution based on RDP bootstrap score >0.8.

## Discussion

Although bulls were previously selected for divergent RFI phenotypes in confinement, RFIg rankings were not consistent with observed RFIc phenotype for all bulls [6]. The re-ranking of RFI phenotypes has been observed with significant dietary changes [23], [24], but these studies do not evaluate RFI under grazing conditions and do not account for differences in grazing selection. While the rumen microbiome is likely partially responsible in determining the RFI phenotype, other factors may collectively have a greater role including tissue metabolism, protein turnover, physical activity, feeding behavior, body composition, and heat increment [2]. Performance testing of bulls in confinement is a commonplace in the beef industry. However, performance evaluation in confined conditions may not be adequate considering a significant proportion of heifer progeny is retained and must utilize forages efficiently to remain in production. Continued efforts to describe the relationship between efficiency under different dietary conditions will improve the utility to producers and decrease production inputs.

In agreement with previous findings [8], [25], two key differences in bacterial taxa were observed between the rumen microbiomes of n-RFI and p-RFI bulls. Fermentation differences have also been detected in divergent RFI phenotypes on similar diets including greater apparent DM and N digestion and greater ruminal ammonia levels in n-RFI cattle [8], [26], [27]. Although the rumen microbiome composition has not been causatively linked to divergent RFI phenotypes, several of the following factors could hinder detection of a relationship: 1) there can be substantial animal-to-animal variation in the rumen bacterial community, so a greater number of animals may be required to observe an association between the rumen microbiome and RFI phenotypes [28], [29]; 2) microbiomes may have similar compositions yet differ in overall microbial metabolic activity potentially causing changes in fermentation; 3) pyrosequencing results only describe relative abundance of bacterial populations and fail to describe quantitative differences in absolute abundance; 4) greater sequencing depth may be needed to detect smaller changes in the rumen microbiome; and 5) RFI rankings within a group may not remain consistent over an animal's lifespan, different diets, and intake measurement methods [26].

There is limited research evaluating effects of different grazing strategies on the rumen microbiome. Although no SI main effects were observed, distinct differences in FA (0.17 and 1.07 kg/kg for HSTK and LSTK, respectively) and stocking rate (15.3 and 6.2 animal units/ha for HSTK and LSTK, respectively; one animal unit  = 365 kg) had a significant impact on ADG [6]. The DMI for bulls on LSTK was 125% greater and may have contributed to observed tendencies for greater richness and diversity. Quantitative approaches should be taken to determine effects on absolute abundance of rumen bacterial populations. The limited FA in HSTK had a greater effect on the rumen microbiome of p-RFI bulls.

Similar to our findings, p-RFI dairy cows consuming fresh ryegrass (23% CP, 36% NDF) had a tendency for a greater relative abundance of *Prevotellaceae* (23.7% vs. 20.8) compared with n-RFI cows [8]. Moreover, *Fibrobacteraceae*and *Entodinium* weregreater in p-RFI cows, while *Lachnospiraceae*and *Dasytricha*were greater in n-RFI cows.However, principal coordinate analysis indicated rumen microbial communities were very similar overall. Beef heifers were evaluated for RFI in confinement and on pasture while qPCR was utilized to monitor changes in the bacterial community; *Prevotella* represented a greater proportion in p-RFI heifers and was the only bacteria related to RFI phenotype [24]. Another study found inconclusive differences between high and low RFI groups using community fingerprinting techniques (DGGE), suggesting greater sequencing depth may be required to discern subtle differences in the rumen microbiome [25].

Cattle consuming bermudagrass hay (6% CP) hadsimilar proportions of *Prevotella* matching the overall average (26%) we observed [30]. Results from the rumen microbiome of dairy cows consuming perennial ryegrass (24% CP, 46% NDF) also indicated *Prevotellaceae* was the predominant family (ranging from 19–23% in relative abundance), while 7–8% sequences were characterized by an unclassified *Bacteroidales* family and *Ruminococcaceae* [31].

In many studies, *Prevotella* has been the dominant observed bacterial genus in the rumen microbiome [30], [32], [33]. The four characterized rumen *Prevotella* species include *P. ruminicola*, *P. bryantii*, *P. albensis*, and *P. brevis* [34], [35]. Even though *Prevotella* may be the most abundant genus observed, cultured species only accounted for a small fraction of the total *Prevotella* population [33], [35]. Cultivated *Prevotella* strains display highly varied genetic divergence [36] suggesting uncultured *Prevotella* also have diverse functions. *Prevotella* species have a documented role in metabolism of starch, hemicellulose, pectin, and peptide or protein catabolism [37]–[40].The whole genome of a *P. ruminicola* and *P. bryantii* strain were sequenced to evaluate their genetic similarity [41]. Of the approximately 40 local syntenic blocks (groups of four or more genes) in each genome, only 14 were shared between the species; most syntenic blocks were associated with polysaccharide metabolism and transport enzymes. Banding patterns from denaturing gradient gel electrophoresis indicated n-RFI and p-RFI steers were each associated with their own *Prevotella*pylotypes [42]. Our core microbiome results from each RFI phenotype also suggest particular *Prevotella* OTUs may be associated with efficient or inefficient cattle and may have different functional roles within the rumen microbiome.

Although Ruminococci have been classically-studied in many culture-based experiments [43], [44], it has been observed in low abundance in the rumen microbiome using molecular techniques [33], [45], [46]. Only 28% of the sequences classified in the family *Ruminococcaceae*were identified in the genus *Ruminococcus*. Two culturable species, *R. flavefaciens* and *R. callidus*, have been associated with cellulose and hemicellulose hydrolysis [47]. Members of *Ruminococcaceae* unidentified at the genus level in this study may be genetically different from cultured ruminococci and possess non-cellulolytic functions.

In conclusion, *Prevotella* was the genus observed in the greatest relative abundance and was greater in the rumen microbiome of p-RFI bulls. High forage allowance and resultant low stocking intensity tended to increase richness and alpha diversity within the rumen microbiome. Our results suggested rumen bacteria communities were largely similar between n-RFI and p-RFI Brahman bulls on HSTK and LSTK, though our sampling size and sequencing depth may have been a limiting factor to observing differences. Future isolation efforts and whole-genome sequencing of uncultured bacteria will provide greater understanding of their metabolic capabilities. Further defining the relationship between the rumen microbiome and efficient animals will allow for additional gains in livestock performance and productivity.

## Supporting Information

Table S1 **Core microbiome OTUs detected in all n-RFI bulls.**
(DOCX)

Table S2 **Core microbiome OTUs detected in all p-RFI bulls.**
(DOCX)
