# Supplementary material for: Relationship between the Rumen Microbiome and Residual Feed Intake-Efficiency of Brahman Bulls Stocked on Bermudagrass Pastures
Source: PLoS One. 2014 Mar 18;9(3):e91864. doi: 10.1371/journal.pone.0091864 (PMC3958397; doi:10.1371/journal.pone.0091864)
Supplement: Table S1 — Core microbiome OTUs detected in all n-RFI bulls. (DOCX) [file pone.0091864.s001.docx]

| **Table S1. Core microbiome OTUs detected in all n-RFI bulls.^1^** | | | | | |
| --- | --- | --- | --- | --- | --- |
| Phylum | Class | Order | Family | Genus | Species |
| *Bacteroidetes* | *Bacteroidia* | *Bacteroidales* |  |  |  |
| *Bacteroidetes* | *Bacteroidia* | *Bacteroidales* |  |  |  |
| *Bacteroidetes* | *Bacteroidia* | *Bacteroidales* |  |  |  |
| *Bacteroidetes* | *Bacteroidia* | *Bacteroidales* |  |  |  |
| *Bacteroidetes* | *Bacteroidia* | *Bacteroidales* |  |  |  |
| *Bacteroidetes* | *Bacteroidia* | *Bacteroidales* |  |  |  |
| *Bacteroidetes* | *Bacteroidia* | *Bacteroidales* |  |  |  |
| *Bacteroidetes* | *Bacteroidia* | *Bacteroidales* |  |  |  |
| *Bacteroidetes* | *Bacteroidia* | *Bacteroidales* |  |  |  |
| *Bacteroidetes* | *Bacteroidia* | *Bacteroidales* |  |  |  |
| *Bacteroidetes* | *Bacteroidia* | *Bacteroidales* |  |  |  |
| *Bacteroidetes* | *Bacteroidia* | *Bacteroidales* |  |  |  |
| *Bacteroidetes* | *Bacteroidia* | *Bacteroidales* |  |  |  |
| *Bacteroidetes* | *Bacteroidia* | *Bacteroidales* |  |  |  |
| *Bacteroidetes* | *Bacteroidia* | *Bacteroidales* | *Paraprevotellaceae* | CF231 |  |
| *Bacteroidetes* | *Bacteroidia* | *Bacteroidales* | *Porphyromonadaceae* | *Paludibacter* |  |
| *Bacteroidetes* | *Bacteroidia* | *Bacteroidales* | *Prevotellaceae* | *Prevotella* | *ruminicola* |
| *Bacteroidetes* | *Bacteroidia* | *Bacteroidales* | *Prevotellaceae* | *Prevotella* |  |
| *Bacteroidetes* | *Bacteroidia* | *Bacteroidales* | *Prevotellaceae* | *Prevotella* |  |
| *Bacteroidetes* | *Bacteroidia* | *Bacteroidales* | *Prevotellaceae* | *Prevotella* |  |
| *Bacteroidetes* | *Bacteroidia* | *Bacteroidales* | *Prevotellaceae* | *Prevotella* |  |
| *Bacteroidetes* | *Bacteroidia* | *Bacteroidales* | *Prevotellaceae* | *Prevotella* |  |
| *Bacteroidetes* | *Bacteroidia* | *Bacteroidales* | *Prevotellaceae* | *Prevotella* |  |
| *Bacteroidetes* | *Bacteroidia* | *Bacteroidales* | *Prevotellaceae* | *Prevotella* |  |
| *Bacteroidetes* | *Bacteroidia* | *Bacteroidales* | *Prevotellaceae* | *Prevotella* |  |
| *Bacteroidetes* | *Bacteroidia* | *Bacteroidales* | *Prevotellaceae* | *Prevotella* |  |
| *Bacteroidetes* | *Bacteroidia* | *Bacteroidales* | *Prevotellaceae* | *Prevotella* |  |
| *Firmicutes* | *Clostridia* | *Clostridiales* | *Clostridiaceae* |  |  |
| *Firmicutes* | *Clostridia* | *Clostridiales* | *Ruminococcaceae* | *Ruminococcus* |  |
| *Firmicutes* | *Clostridia* | *Clostridiales* | *Ruminococcaceae* |  |  |
| *Firmicutes* | *Clostridia* | *Clostridiales* | *Ruminococcaceae* |  |  |
| *Firmicutes* | *Clostridia* | *Clostridiales* | *Ruminococcaceae* |  |  |
| *Firmicutes* | *Clostridia* | *Clostridiales* | *Ruminococcaceae* |  |  |
| *Firmicutes* | *Clostridia* | *Clostridiales* | *Veillonellaceae* | *Succiniclasticum* |  |
| *Firmicutes* | *Clostridia* | *Clostridiales* |  |  |  |
| *Lentisphaerae* | *Lentisphaeria* | *Victivallales* | *Victivallaceae* |  |  |
| *Lentisphaerae* | *Lentisphaeria* | *Victivallales* | *Victivallaceae* |  |  |
| *Lentisphaerae* | *Lentisphaeria* | *Victivallales* | *Victivallaceae* |  |  |
| *Proteobacteria* | *Alphaproteobacteria* |  |  |  |  |

^1^OTUs = operational taxonomic units; taxonomic classification is given at the greatest taxonomic resolution based on RDP bootstrap score > 0.8.
